# Publisher Correction: Data-driven acceleration of photonic simulations

**DOI:** 10.1038/s41598-020-59308-5

**Published:** 2020-02-19

**Authors:** Rahul Trivedi, Logan Su, Jesse Lu, Martin F. Schubert, Jelena Vuckovic

**Affiliations:** 1 E. L. Ginzton Laboratory, Stanford University, Stanford, CA 94305 USA; 2 X, Mountain View, CA 94043 USA

Correction to: *Scientific Reports* 10.1038/s41598-019-56212-5, published online 23 December 2019

This Article contains an error in the order of the Figures.

Figure 1 was incorrectly published as Figure 3.

Figure 2 was incorrectly published as Figure 1.

Figure 3 was incorrectly published as Figure 2.

The correct Figures are reproduced below with their corresponding legends.

**Figure 1:**
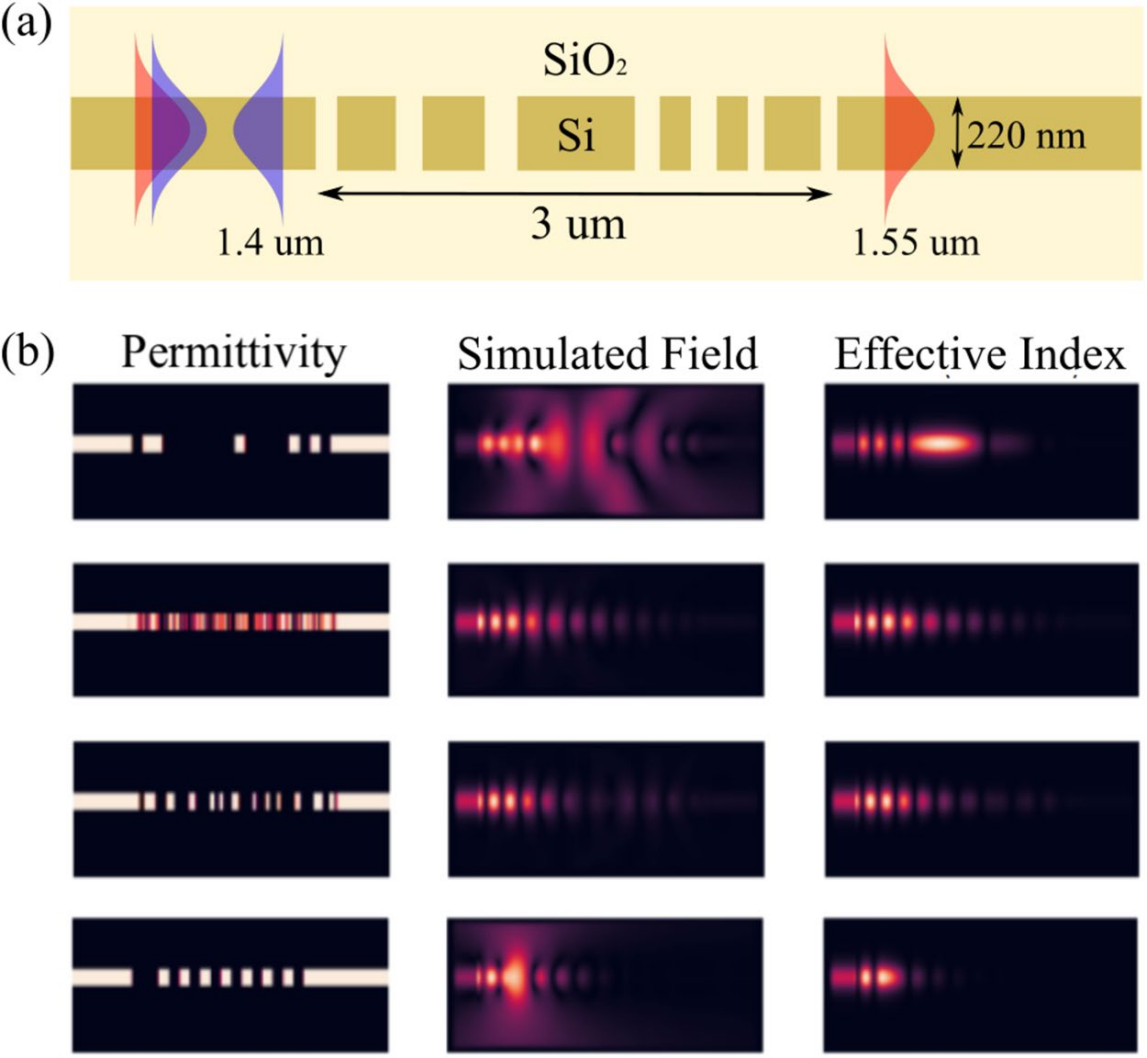
(**a**) Schematic of the grating splitter device that comprises the dataset. All the gratings in the dataset are 3 *μ*m long and are designed for a 220 nm silicon-on-insulator (SOI) platform with oxide cladding. We use a uniform spatial discretization of 20 nm while representing Eq. 1 as a system of linear equations. The resulting system of linear equations has 229 × 90 = 20,610 unknown complex numbers. (**b**) Visualizing samples from the dataset — shown are permittivity distribution, simulated electric fields and effective index fields for 4 randomly chosen samples. All fields are shown at a wavelength of 1.4 *μ*m.

**Figure 2:**
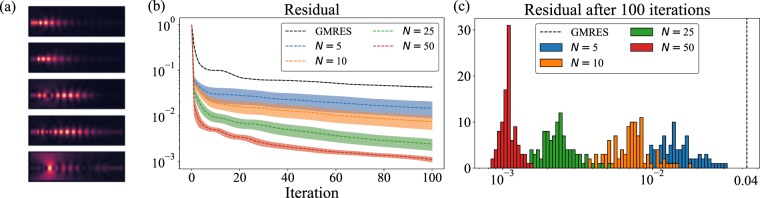
(**a**) First five principal components of the electric fields in the grating splitter dataset. (**b**) Performance of data-driven GMRES on the evaluation dataset when supplied with different number of principal components (~200 samples from the training set were used for computing the principal components) — the dotted line shows the mean residual, and the solid colored background indicates the region within one standard deviation around the mean residual. (**c**) Histogram of the residual after 100 data-driven GMRES iterations for different *N* computed over 100 randomly chosen samples from the evaluation dataset. The black vertical dashed line indicates the mean residual after 100 iterations of GMRES over the evaluation dataset.

**Figure 3:**
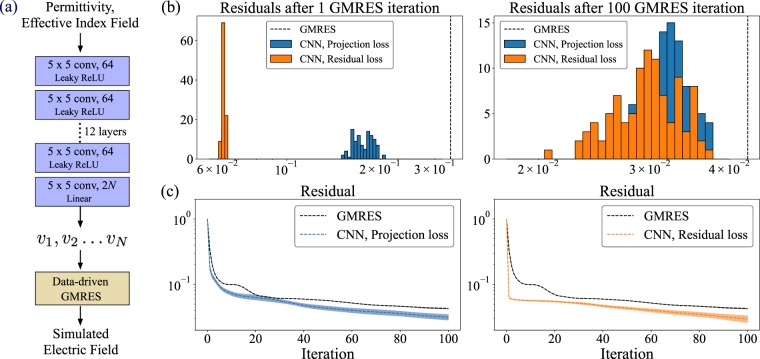
(**a**) Schematic of the CNN based data-driven GMRES — a convolutional neural network takes as input the permittivity and effective index field and produces as an output the vectors *v*1, *v*2 … *vN*. These vectors are then supplied to the data-driven GMRES algorithm, which produces the full simulated field. (**b**) Histogram of the residual after 1 and 100 data-driven GMRES iterations evaluated over the evaluation dataset. We consider neural networks trained with both the projection loss function *l*proj and residual loss function *l*res. The vertical dashed lines indicate the mean residual after 1 and 100 iterations of GMRES over the evaluation dataset. (**c**) Performance of the data-driven GMRES on the evaluation dataset when supplied with the vectors at the output of the convolutional neural networks trained with the projection loss function *l*proj and the residual loss function *l*res. The dotted line shows the mean residual, and the solid colored background indicates the region within ± standard deviation around the mean residual.

