# Supplementary material for: Data-driven acceleration of photonic simulations
Source: Sci Rep. 2019 Dec 23;9:19728. doi: 10.1038/s41598-019-56212-5 (PMC6928023; doi:10.1038/s41598-019-56212-5)
Supplement: Supplementary file 1 — Supplementary Information [file 41598_2019_56212_MOESM1_ESM.pdf]

Supplementary to 'Data-driven acceleration of photonic simulations'

Rahul Trivedi[1,2,*], Logan Su[1], Jesse Lu[2], Martin F. Schubert[2], and Jelena Vuckovic[1]

[1]E. L. Ginzton Laboratory, Stanford University, Stanford, CA 94305, USA
[2]X, Mountain View, CA 94043, USA
[*]rtrivedi@stanford.edu

1 Data-driven GMRES

By following the same steps as in GCROT (Generalized Conjugate Residual with inner Orthogonalization adn outer Truncation), an efficient update rule can be developed for the data-driven GMRES iteration (defined by Eq. 3 of the main text). In this section, we provide more details on the derivation of this update rule.

Notation and prelimnaries:

1. The system of equations being solved will be denoted by $Af = b$, with f being the unknown vector being solved for. We also denote by D the size of the system of equations i.e. $A \in \mathbb{C}^{D\times D}$ and $f, b \in \mathbb{C}^D$. It will be assumed that A is invertible.

2. Given the vectors $v_1, v_2 \dots v_N$ with which GMRES has to be accelerated (which are assumed to be linearly independent, but not necessarily orthogonal), we will denote by V the matrix that is formed with these vectors as its columns. Note that $V \in \mathbb{C}^{D\times N}$ and $\text{span}(v_1, v_2 \dots v_N) = \text{range}(V)$.

3. $\mathcal{K}_n(A, b)$ will denote the Krylov subspace of dimensionality n that is generated by the matrix A and the vector b: $\mathcal{K}_n(A, b) = \text{span}(b, Ab, A^2b \dots A^{n-1}b)$.

4. $\tilde{A}$ and $\tilde{b}$ are defined by:

$$\tilde{A} = P_\perp(Av_1, Av_2 \dots Av_N)A \tag{1a}$$

$$\tilde{b} = P_\perp(Av_1, Av_2 \dots Av_N)b \tag{1b}$$

   where $P_\perp(Av_1, Av_2 \dots Av_N)$ is the operator projecting a vector out of $\text{span}(Av_1, Av_2 \dots Av_N)$. For convenience, we will denote this operator by just $P_\perp$. We note that, in general, $\tilde{A}$ is not sparse even if A is sparse, but for small N multiplication of $\tilde{A}$ with a vector can be computed efficiently by first multiplying the vector by A, followed by projecting the resulting vector out of $\text{span}(Av_1, Av_2 \dots Av_N)$.

5. To conveniently work with $P_\perp$, we perform an incomplete QR decomposition on the matrix AV to obtain an orthogonal matrix $C \in \mathbb{C}^{D\times N}$ and an upper triangular matrix $R \in \mathbb{C}^{N\times N}$: $AV = CR$. It then immediately follows that $P_\perp = \mathrm{I} - CC^\dagger$. Moreover, it is also convenient to precompute and store R^{-1} (Note that if A is invertible, and $v_1, v_2 \dots v_N$ are linearly independent then R is invertible).

Arnoldi iteration: The ith iteration of data-driven GMRES approximates the solution to $Af = b$ with f_i, where f_i is given by:

$$f_i = \underset{f\in\text{range}(V)\oplus\mathcal{K}_i(\tilde{A},\tilde{b})}{\text{argmin}} ||Af - b||^2 \tag{2}$$

One of the key ingredients of the GMRES iteration is the Arnoldi iteration which generates an orthonormal basis for the Krylov subspace $\mathcal{K}_{i+1}(\tilde{A}, \tilde{b})$ from the orthonormal basis for the Krylov subspace $\mathcal{K}_i(\tilde{A}, \tilde{b})$. Denoting the orthonormal basis for $K_i(\tilde{A}, \tilde{b})$ by $\{q_1, q_2 \dots q_i\}$, note that $\text{span}(q_1, q_2 \dots q_i, \tilde{A}q_i) = \mathcal{K}_{i+1}(\tilde{A}, \tilde{b})$. Therefore, q_{i+1} can be computed by orthonormalizing $\tilde{A}q_i$ against $\{q_1, q_2 \dots q_i\}$:

$$q_{i+1} = \frac{v_{i+1}}{||v_{i+1}||}, \text{ where } v_{i+1} = \tilde{A}q_i - \sum_{j=1}^{i} (q_j^\dagger \tilde{A} q_i) q_j \tag{3}$$

In our implementation, we assume $q_1 = \tilde{b}/||\tilde{b}||$, and use Eq. 3 to generate $q_2, q_3 \dots$ and so on. Note that $q_i \perp \text{span}(Av_1, Av_2 \dots Av_N)\ \forall\ i$, or equivalently $C^\dagger Q_i = 0\ \forall\ i$. Denoting by Q_i the matrix formed with the vectors $q_1, q_2 \dots q_i$ as its columns ($Q_i \in \mathbb{C}^{D\times i}$), the Arnoldi iteration can be expressed as the following relationship between Q_{i+1} and Q_i:

$$\tilde{A}Q_i = Q_{i+1}H_{i,i+1} \implies AQ_i = Q_{i+1}H_{i,i+1} + CC^\dagger AQ_i \tag{4}$$

where $H_{i,i+1} \in \mathbb{C}^{(i+1)\times i}$ is an upper Hessenberg matrix defined by:

$$H_{i,i+1} = \begin{bmatrix} q_1^\dagger \tilde{A} q_1 & q_1^\dagger \tilde{A} q_2 & q_1^\dagger \tilde{A} q_3 & \dots & q_1^\dagger \tilde{A} q_i \\ ||v_2|| & q_2^\dagger \tilde{A} q_2 & q_2^\dagger \tilde{A} q_3 & \dots & q_2^\dagger \tilde{A} q_i \\ 0 & ||v_3|| & q_3^\dagger \tilde{A} q_3 & \dots & q_3^\dagger \tilde{A} q_i \\ 0 & 0 & ||v_4|| & \dots & q_4^\dagger \tilde{A} q_i \\ \vdots & \vdots & \vdots & \ddots & \vdots \\ 0 & 0 & 0 & \dots & ||v_{i+1}|| \end{bmatrix} \tag{5}$$

Calculating f_i: Consider now solving the optimization problem in Eq. 2. Since the optimization variable f is in the space $\text{range}(V) \oplus \mathcal{K}_i(\tilde{A}, \tilde{b})$, it can be expressed as:

$$f = VR^{-1}x + Q_i y \tag{6}$$

where $x \in \mathbb{C}^N$ and $y \in \mathbb{C}^i$. Thus, it follows that:

$$\begin{aligned} ||Af - b||^2 &= ||AVR^{-1}x + AQ_i y - b||^2 \\ &= \left|\left| [C\ Q_{i+1}] \begin{bmatrix} I & C^\dagger AQ_i \\ 0 & H_{i,i+1} \end{bmatrix} \begin{bmatrix} x \\ y \end{bmatrix} - b \right|\right|^2 \end{aligned} \tag{7}$$

wherein we have used $AV = CR$ and Eq. 4. Note that since C and Q_{i+1} are independently orthogonal matrix, and $C^\dagger Q_{i+1} = 0$, it follows that $[C\ Q_{i+1}]$ is an orthogonal matrix. Eq. 7 can now be further simplified to:

$$||Af - b||^2 = \left|\left| \begin{bmatrix} I & C^\dagger AQ_i \\ 0 & H_{i,i+1} \end{bmatrix} \begin{bmatrix} x \\ y \end{bmatrix} - \begin{bmatrix} C^\dagger b \\ Q_{i+1}^\dagger b \end{bmatrix} \right|\right|^2 + ||(\mathrm{I} - CC^\dagger - Q_{i+1}Q_{i+1}^\dagger)b||^2 \tag{8}$$

Therefore, $f_i = VR^{-1}x_i + Q_i y_i$, where

$$x_i, y_i = \underset{x,y}{\text{argmin}} \left|\left| \begin{bmatrix} I & C^\dagger AQ_i \\ 0 & H_{i,i+1} \end{bmatrix} \begin{bmatrix} x \\ y \end{bmatrix} - \begin{bmatrix} C^\dagger b \\ Q_{i+1}^\dagger b \end{bmatrix} \right|\right|^2 \tag{9}$$

We have thus reduced the problem of calculating f_i, which was a constrained least squares problem, to an unconstrained least squares problem (Eq. 9) of size $i + N$, which can be solved numerically (e.g. using QR factorization).

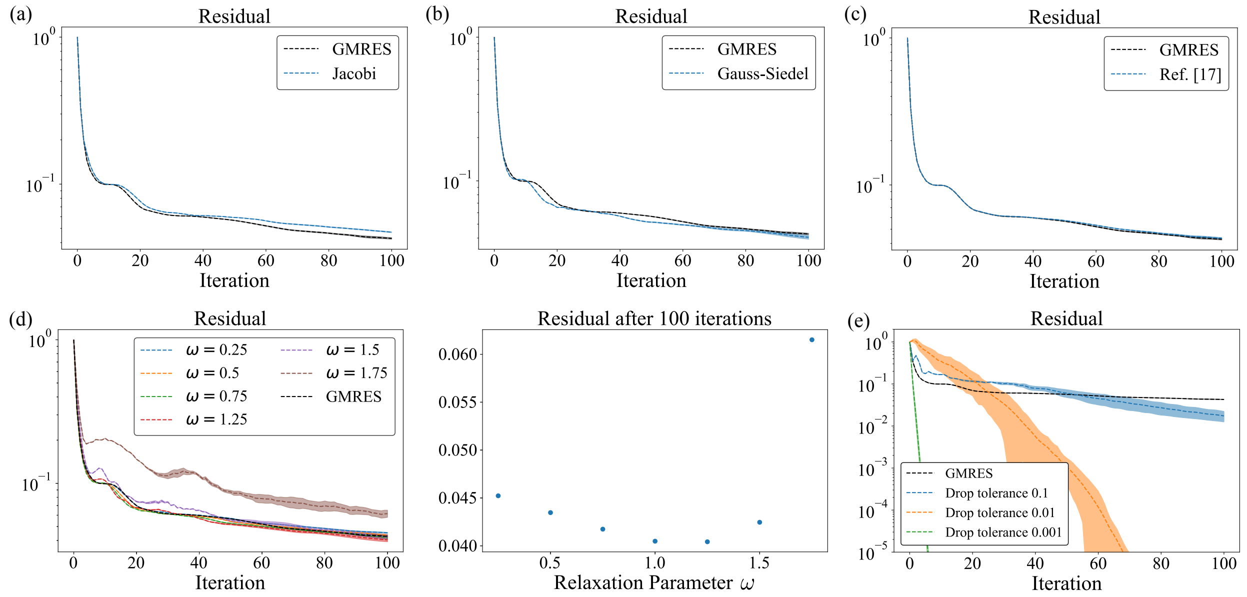

Figure 1: Performance of the different preconditioners described in section 2 on the evaluation dataset. (a) Jacobi preconditioner, (b) Gauss-Siedel preconditioner, (c) preconditioner from ref. [17] of main text (d) Symmetric over-relaxation (SOR) preconditioner for different relaxation parameter ω and (e) Incomplete LU preconditioner for different drop tolerances.

2 Benchmarks for data-free preconditioners

Here we present the results of applying some data-free preconditioners on the simulation problem. Given a left preconditioner P_L and a right preconditioner P_R, the system of equations being solved is transformed from $Af = b$ to $A'f' = b'$ where:

$$A' = P_L A P_R,\ b' = P_L b \text{ and } f' = P_R^{-1} f \tag{10}$$

We study the following four preconditioners:

1. *Jacobi preconditioner*: The Jacobi preconditioner [1] is given by:

$$P_L = \mathcal{D}(A)^{-1} \text{ and } P_R = I \tag{11}$$

   where $\mathcal{D}(A)$ is a diagonal matrix formed from the diagonal entries of the matrix A. The performance of Jacobi preconditioner on the evaluation dataset is shown in Fig. 1(a).

2. *Gauss-Siedel preconditioner*: The Gauss-Siedel preconditioner is given by:

$$P_L = [\mathcal{D}(A) + \mathcal{L}(A)]^{-1} \text{ and } P_R = I \tag{12}$$

   where $\mathcal{L}(A)$ is a strictly lower-triangular matrix formed by the elements of A below the main diagonal. Note that application of this preconditioner requires the solution a lower triangular system of equations. The performance of the Gauss-Siedel preconditioner on the evaluation dataset is shown in Fig. 1(b).

3. *Preconditioner from ref. [17]*: This preconditioner is specific to Maxwell's equations. P_R and P_L are diagonal matrices constructed from the grid spacing (including the complex stretching due to PMLs) in the simulation domain. Details of this preconditioner can be found in ref. [17] for main text. The performance of this preconditioner on the evaluation dataset is shown in Fig. 1(c).

4. *Symmetric over-relaxation (SOR) preconditioner*: The SOR preconditioner [1] is given by:

$$P_L = [\mathcal{D}(A) + \omega\mathcal{L}(A)]^{-1} \text{ and } P_R = I \tag{13}$$

where $\mathcal{L}(A)$ is a strictly lower-triangular matrix formed by the elements of A below the main diagonal and ω is a tunable parameter (referred to as the relaxation parameter) in the preconditioner which can be between 0 to 2. Note that the SOR preconditioner for $\omega = 1$ is identical to the Gauss-Siedel preconditioner. Additionally, application of the SOR preconditioner requires the solution of a lower triangular system of equations. The performance of the SOR preconditioner on the evaluation dataset is shown in Fig. 1(d) — we see that the best performance of SOR preconditioner on our dataset is achieved for $\omega = 1.25$.

5. *Incomplete LU*: This preconditioner seeks an upper and lower triangular matrix, U and L such that the product LU is approximately equal to the matrix A [1]. The preconditioner is then given by:

$$P_L = U^{-1}L^{-1} \text{ and } P_R = I \tag{14}$$

The deviation of the LU from A is controlled with a drop tolerance parameter — a larger drop tolerance implies a faster computation of L and U but a worse approximation to A and therefore a less useful preconditioner. The performance of incomplete LU preconditioner on the evaluation dataset is shown in Fig. 1(e) for drop tolerances of $0.1, 0.01$ and 0.001.

References

[1] Yousef Saad. *Iterative methods for sparse linear systems*, volume 82. siam, 2003.
